# Added value of autoregulation and multi-step kinetics of transcription initiation

**DOI:** 10.1098/rsos.181170

**Published:** 2018-11-28

**Authors:** Mahendra Kumar Prajapat, Andre S. Ribeiro

**Affiliations:** 1 Laboratory of Biosystem Dynamics, Faculty of Biomedical Sciences and Engineering, BioMediTech Institute, Tampere University of Technology, 33101 Tampere, Finland; 2 Multi-scaled Biodata Analysis and Modelling Research Community, Tampere University of Technology, 33101 Tampere, Finland; 3 CA3 CTS/UNINOVA, Faculdade de Ciencias e Tecnologia, Universidade Nova de Lisboa, Quinta da Torre, 2829-516 Caparica, Portugal

**Keywords:** transcription initiation, rate-limiting steps, cell-to-cell variability, autoregulation mechanisms

## Abstract

Bacterial gene expression regulation occurs mostly during transcription, which has two main rate-limiting steps: the close complex formation, when the RNA polymerase binds to an active promoter, and the subsequent open complex formation, after which it follows elongation. Tuning these steps' kinetics by the action of e.g. transcription factors, allows for a wide diversity of dynamics. For example, adding autoregulation generates single-gene circuits able to perform more complex tasks. Using stochastic models of transcription kinetics with empirically validated parameter values, we investigate how autoregulation and the multi-step transcription initiation kinetics of single-gene autoregulated circuits can be combined to fine-tune steady state mean and cell-to-cell variability in protein expression levels, as well as response times. Next, we investigate how they can be jointly tuned to control complex behaviours, namely, time counting, switching dynamics and memory storage. Overall, our finding suggests that, in bacteria, jointly regulating a single-gene circuit's topology and the transcription initiation multi-step dynamics allows enhancing complex task performance.

## 1. Introduction

Bacterial cells can tune their gene expression profile in response to environmental changes [1–8]. E.g. in *Escherichia coli*, this adaptability is made possible by, among other things, fine-tuning the transcription kinetics of its genes [9]. This is enhanced by the multi-step nature of transcript initiation [10–13], whose steps can be individually or jointly controlled by promoter-specific external signals (e.g. transcription factors), global regulators such as *σ* factors, etc. [10,14–19]. Evidence suggests that both the mean rate and noise in this dynamics can be tuned [20].

One way to halt a promoter's activity is the intervention of a transcription factor (TF), capable of negative regulation [11,21]. Other transcription factors can act as activators [10,21,22]. Relevantly, the transcription dynamics is sequence dependent because e.g. the promoter sequence affects the kinetics of the rate-limiting steps in the initiation, altering the mean and cell-to-cell variability in RNA, and thus, protein numbers [15,18,23–25].

Almost 60% of the TFs produced by *E. coli* are autoregulators [26–28]. Autoregulation allows genes to behave as molecular clocks, switches or memory storage units that assist cells in better controlling response levels and times, etc. [29]. The outcome of introducing TFs in the cytoplasm is usually a change in the kinetics of the rate-limiting steps in transcription initiation of a specific gene(s) [11]. The result of this intervention depends on which rate-limiting step(s) is affected. Namely, acting on a longer-lasting step is likely to have a stronger effect on the RNA production rate than when acting on the shorter-lasting step [23].

In autoregulated genes, the regulatory mode (repression versus activation and the strength of the regulation) is of importance in the resulting changes in mean and cell-to-cell variability in protein numbers [15,30–34], as these affect the fitness of microbial populations [1,9,35,36]. Based on past knowledge (see e.g. [23]) we predict that the effects of autoregulation depend not only on the mode and strength of this regulation but also on the dynamics of transcription initiation of the gene under regulation.

Here, using parameter values extracted from recent single-cell measurements of transcription and translation kinetics in live cells, we designed stochastic models of gene expression controlled by different regulatory modes to explore how the combination of regulation by the action of TFs and regulation of the rate-limiting steps in transcription initiation expands the state space of possible behaviours of autoregulated and externally regulated single-gene circuits. For this, we perform stochastic simulations for varying inducer concentrations, relative durations of the rate-limiting steps in transcription initiation, and binding strengths of the activator/repressor transcription factors (to tune the feedback strength in autoregulation). From these simulations, we assess the mean expression levels at ‘steady state’ (i.e. after a long time period), the cell-to-cell variability in gene expression products and the response times of our circuit in model cells. We note that the term ‘steady state’ here refers to ‘noisy attractors’ [37] because, technically, stochastic models do not have steady states. Finally, we also implement model constitutive genes as null-models, so as to provide a point of reference for quantifying the effects of the autoregulation mechanisms on the gene expression kinetics.

## 2. Material and methods

### 2.1. Models of gene expression and regulation mechanisms

**Figure 1.**
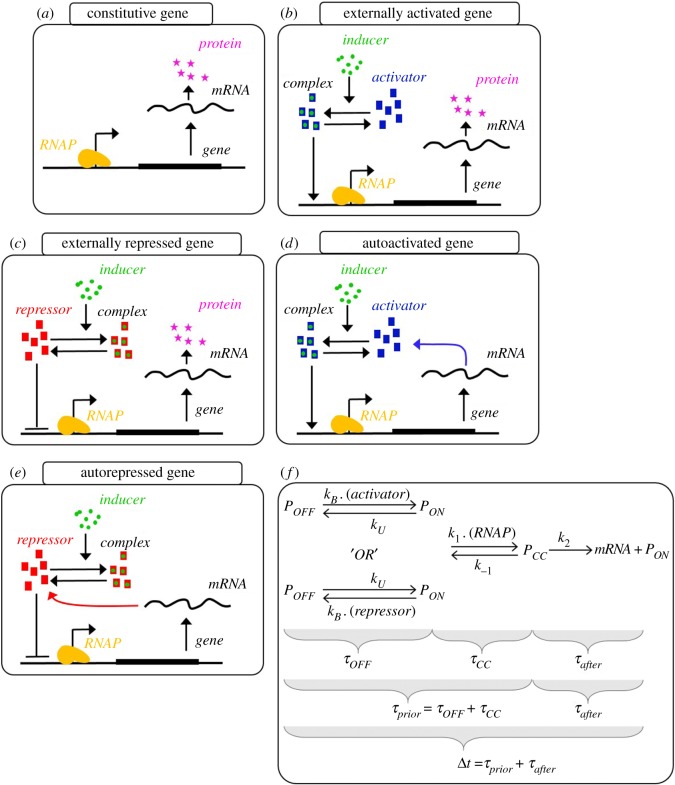
Regulatory modes of a gene expression. (*a*) Constitutive gene, (*b*) externally activated gene, (*c*) externally repressed gene, (*d*) autoactivated gene and (*e*) autorepressed gene. (*f*) Representation of the mean times spent in the rate-limiting steps in transcription initiation. *P*_OFF_, *P*_ON_ and *P*_CC_ represent the promoter in three states (respectively, unavailable for transcription, available for transcription and committed to closed complex formation). These states are controlled by the binding/unbinding of activator/repressor molecules, followed by the binding/unbinding of an RNAP (RNA polymerase) and the kinetics afterwards. Namely, *τ*_OFF_, *τ*_CC_ and *τ*_after_ represent the average time spent in each rate-limiting step, respectively, the ‘OFF’ state (i.e. repressed), the closed complex formation, ‘CC’, and the open complex formation (after), after which an RNA molecule is synthesized. In detail, *τ*_after_ corresponds to the mean time spent following the commitment to open complex formation. Finally, Δ*t* corresponds to the time interval between consecutive RNA production events. In constitutive genes, *τ*_OFF_ ∼ 0.

We model gene expression when constitutive (acting as a ‘null model’) and when externally or autoregulated by activator/repressor TFs, which act on transcription initiation. The models are depicted in figure 1. In all, we assume a multi-step model of active transcription [38,39], validated in [12]. We note that this model should be applicable to plasmid-borne and chromosome-integrated promoters, provided that the latter are not located in highly expressed operons and, thus, are not strongly influenced by promoter halting due to the accumulation of positive supercoiling build-up [20].

From figure 1*a*, constitutive promoters are always active [40,41] (i.e. in the ON state). Thus, their expression rate is regulated by the binding/unbinding rates of RNA polymerases (RNAPs) [41]. Constitutive gene expression levels usually depend mostly on the cell growth rate [17,41–43], as this rate can affect the RNAP concentration.

Meanwhile, promoters subject to regulation by TFs can have their activity reduced/enhanced during the time period when repressors/activators are present in the system [44,45]. For that, we assume that when bound by an activator/repressor their activity is affected accordingly (enhanced/reduced). We note that, given the use of a two-step model of transcription (reactions 2.5 and 2.6 below), this model behaviour is not identical to that of a simple on–off switch. These interactions are modelled, respectively, by reaction 2.1 and reaction 2.2:

2.1 $$P_{\mathrm{OFF}}+\mathrm{activator}\overset{k_{B}}{\underset{k_{U}}{\overset{⟶}{⟵}}}P_{\mathrm{ON}}$$

and

2.2 $$P_{\mathrm{ON}}+\mathrm{repressor}\overset{k_{B}}{\underset{k_{U}}{\overset{⟶}{⟵}}}P_{\mathrm{OFF}}.$$

In reactions 2.1 and 2.2, the unbinding of activators/repressors occurs at the constant rate *k*_U_, while their binding occurs at the rate *k*_B_, which depends on inducer/repressor concentration and their effective binding affinity (*k*_B_) [46] (estimated by equations (2.3) and (2.4)).

The inducer concentration is represented by *I*. The maximum and minimum affinities of activator/repressor to the promoter are represented by, respectively, *C*_max_ and *C*_min_ (see electronic supplementary material in [46]). Meanwhile, *K*_I_ is a half-maximum concentration of inducers for activators (in % w/v) and repressors (in mM). The binding strength of activators/repressors can be tuned by altering factor ‘*f*’, which is the relative ratio of TF binding rates, which we use as a measure of the feedback strength of autoregulated genes.

2.3 $$k_{B}=f\;.\;k_{U}\;.\;\left[(C_{\max}-C_{\min})\;.\;\frac{{\left(I/K_{I}\right)}^{2}}{1+{\left(I/K_{I}\right)}^{2}}+C_{\min}\right]$$

and

2.4 $$k_{B}=f\;.\;k_{U}\;.\;\left[(C_{\max}-C_{\min})\;.\;\frac{1}{1+{\left(I/K_{I}\right)}^{2}}+C_{\min}\right].$$

In all models, transcription starts with the binding of a free RNAP to an active promoter (*P*_ON_), forming a closed complex, *P*_CC_ [10,19,22,47,48] (reaction 2.5). As this step is reversible, multiple closed complex formations can occur between two consecutive RNA production events [12] (reaction 2.5). When ‘successful’, it follows the irreversible open complex formation [10,49,50]. Once complete, an mRNA will be produced and the promoter becomes again available to RNAPs [10,51] (reaction 2.6). In reaction 2.6, *k*_2_ represents the inverse of the mean time-length for the open complex to be complete, once initiated:

2.5 $$P_{\mathrm{ON}}\overset{k_{1}\;.\;\mathrm{RNAP}}{\underset{k_{-1}}{\overset{⟶}{⟵}}}P_{\mathrm{CC}}$$

and

2.6 $$P_{\mathrm{CC}}\overset{k_{2}}{⟶}\mathrm{mRNA}+P_{\mathrm{ON}}.$$

The model does not include transcription elongation nor termination because, in normal conditions, these steps are much faster than the rate-limiting steps in initiation [11,52–59], not affecting significantly the time intervals between RNA production events.

In models where gene expression is regulated by TFs, but feedback reactions are absent (figure 1*b*,*c*), the production of activators/repressors is assumed to occur at a basal rate (reaction 2.7):

2.7 $$\emptyset \overset{k_{\mathrm{basal}}}{\to }\;\frac{\mathrm{Activator}}{\mathrm{Repressor}}.$$

Model autoregulated genes (figure 1*d*,*e*) produce their activators/repressors, which establish a feedback regulatory system (reactions 2.1 and 2.2). Activators/repressors are produced from the RNAs (reaction 2.8):

2.8 $$\mathrm{mRNA}\overset{k_{p}}{⟶}\mathrm{mRNA}+\mathrm{protein}/\mathrm{activator}/\mathrm{repressor}.$$

Finally, we assume constant degradation rates of mRNAs (reaction 2.9) and TFs (reaction 2.10) [60–62]:

2.9 $$\mathrm{mRNA}\;\overset{d_{\mathrm{mRNA}}}{⟶}\emptyset$$

and

2.10 $$a\mathrm{ctivator}/\mathrm{repressor}/\mathrm{protein}\overset{d_{\mathrm{Protein}}}{⟶}\emptyset .$$

The parameter values used here are based on empirical data or have been fitted to physiologically realistic ranges (shown in table 1, unless stated otherwise). Finally, as an approximation, the models assume only one copy of the gene of interest in the cell [12].

**Table 1.** List of parameter values of the models. Shown are their values and the references from which they were gathered. The symbol ‘*’ stands for ‘fitted to achieve physiologically realistic ranges'. The symbol ‘+’ stands for ‘varied within realistic intervals'. w/v stands for weight by volume. We opted for extracting as many rate constants as possible from the same publication(s), for model consistence.

parameter	description	values	refs.
*k*_basal_	basal synthesis rate of activator/repressor	0.07 protein min^−1^	*
*k*_U_	unbinding of activator/repressor from promoter	1.8 min^−1^	[46]
*C*_max_	max. affinity of activators to the binding site	1 molecule^−1^	[46]
	max. affinity of repressors to the binding site	0.2 molecule^−1^	[46]
*C*_min_	min. affinity of activators to the binding site	0 molecule^−1^	[46]
	min. affinity of repressors to the binding site	0.01 molecule^−1^	[46]
*K*_I_	half-maximal concentration of inducer for activator	2.5% (w/v)	[46]
	half-maximal concentration of inducer for repressor	0.035 mM	[46]
*k*_1_	binding affinity of the RNAP to the promoter	+	[12]
*k*_−1_	unbinding affinity of the RNAP from the promoter	60 min^−1^	[12]
*k*_2_	effective rate of events after transcription initiation until mRNA synthesis	+	[12]
*k*_p_	rate of translation	27 protein min^−1^	[63–65]
*d*_mRNA_	rate of mRNA degradation	0.12 min^−1^	[60,66]
*d*_protein_	rate of protein/activator/repressor degradation	0.0231 min^−1^	[67]
RNAP	number of free RNA polymerases	1000	[68–70]
*I*	inducer concentration for activators	[0–2.5] % (w/v)	[23,42,47]
	inducer concentration for repressor	[0–1] mM	[23,42,47]
Δ*t*	avg. duration of transcription intervals	[1–20] min	[12,71–73]

### 2.2. Transcription initiation kinetics and interval between transcription events

In [12], a method was proposed for dissecting the *in vivo* kinetics of the rate-limiting steps in active transcription (figure 1*f*). Shortly, from measurements of intervals between consecutive transcription events in individual cells (Δ*t*) at different RNAP concentrations, one can infer the mean duration of the events *prior* to (*τ*_prior_) and *after* (*τ*_after_) the commitment to open complex formation. This is possible as the value of *τ*_prior_ differs with a concentration of RNAP, while *τ*_after_ does not [10]. Here, for simplicity, we assume that TFs also only affect the kinetics of the first step.

According to the models, the mean interval between consecutive RNA production events (Δ*t*) equals, when regulated by activators or repressors, respectively (equations (2.11) and (2.12)):

2.11 $$\Delta t=\frac{\left(k_{B}+k_{U})(k_{-1}+k_{2}\right)}{\mathrm{RNAP}\cdot k_{1}\cdot k_{2}\cdot k_{B}}+\frac{1}{k_{2}}$$

and

2.12 $$\Delta t=\frac{\left(k_{B}+k_{U})(k_{-1}+k_{2}\right)}{\mathrm{RNAP}\cdot k_{1}\cdot k_{2}\cdot k_{U}}+\frac{1}{k_{2}}.$$

In constitutively expressed genes, Δ*t* equals (equation (2.13)):

2.13 $$\Delta t=\frac{\left(k_{-1}+k_{2}\right)}{\mathrm{RNAP}\cdot k_{1}\cdot k_{2}}+\frac{1}{k_{2}}.$$

### 2.3. Simulations and dynamics evaluation

To simulate the models, we use SGNSim (Stochastic Gene Networks Simulator) [74], a simulator of chemical reaction systems according to the Gillespie's stochastic simulation algorithm [75,76]. In addition, this simulator allows for the reaction rates to be calculated from complex functions or from physical parameters, when necessary. SGNSim was designed to e.g. model specific genetic circuits and systems of chemical reactions. It further allows for perturbations during simulations, including the introduction of new components in the system.

We focus on how tuning the kinetics of transcription initiation affects the behaviour of the model circuits. For this, instead of changing the mean transcription rate, we alter the fraction of time spent in the events prior to and after initiation of the open complex formation. Namely, we vary *τ*_after_/Δ*t* between 0.05 and 0.95, to cover the wide diversity of empirical values reported in [13,18]. For this, Δ*t* is kept constant, and *k*_1_ and *k*_2_ are changed according to equations (2.12)–(2.15), depending on the circuit's topology. By keeping Δ*t* constant, the RNA production kinetics (e.g. its noise) is changed due to changing the quantitative relationship between *τ*_prior_ and Δ*t*, rather than due to changing the mean rate of transcription (which would require changing Δ*t*). This is because changes in Δ*t* are limited by biophysical constraints such as intracellular concentration of RNA polymerases, promoter affinity biophysical limitations, etc. while empirical evidence suggests that *τ*_prior_/Δ*t* can be changed from almost 0 to almost 1 [13,18].

The rates *k*_1_ and *k*_2_ for constitutive genes were estimated using equations (2.14) and (2.15). Since, in this model, the kinetics of the steps following initiation of the open complex formation does not depend on the regulatory molecules, *k*_2_ of externally regulated genes (thus, without feedback) is also estimated using equation (2.15).

2.14 $$k_{1}=\frac{\Delta t\cdot k_{-1}\;\;-\;\Delta t\cdot (\tau_{\mathrm{prior}}/\Delta t)\cdot k_{-1}\;+\;1}{\Delta t\cdot \mathrm{RNAP}\cdot (\tau_{\mathrm{prior}}/\Delta t)}\;$$

and

2.15 $$k_{2}=\frac{1}{\Delta t\;.\;[1-(\tau_{\mathrm{prior}}/\Delta t)]}.$$

Meanwhile, *k*_1_ depends on the inducer and TF intracellular concentrations and, thus, is calculated using equation (2.16) for positive regulation and equation (2.17) for negative regulation. To change the induction strength, while maintaining Δ*t* and *τ*_prior_/Δ*t* constant, we change *k*_1_, in accordance with equation (2.16) (for activation) and equation (2.17) (for repression), for both externally regulated and self-regulated genes.

2.16 $$k_{1}=\frac{k_{B}\;+\;k_{U}\;+\;\Delta t\cdot k_{U}\cdot k_{-1}\;-\;\Delta t.(\tau_{\mathrm{prior}}/\Delta t)\;.\;k_{U}\cdot k_{-1}\;+\;\Delta t\cdot k_{B}.k_{-1}\;-\;\Delta t\cdot (\tau_{\mathrm{prior}}/\Delta t)\cdot k_{B}\cdot k_{-1}}{\Delta t\cdot \mathrm{RNAP}\cdot k_{B}\cdot (\tau_{\mathrm{prior}}/\Delta t)}$$

and

2.17 $$k_{1}=\frac{k_{B}\;+\;k_{U}\;+\;\Delta t\cdot k_{U}\cdot k_{-1}\;-\;\Delta t\cdot (\tau_{\mathrm{prior}}/\Delta t)\cdot k_{U}\cdot k_{-1}\;+\;\Delta t.k_{B}.k_{-1}\;-\;\Delta t\cdot (\tau_{\mathrm{prior}}/\Delta t)\cdot k_{B}\cdot k_{-1}}{\Delta t\cdot \mathrm{RNAP}\cdot k_{U}\cdot (\tau_{\mathrm{prior}}/\Delta t)}.$$

For any given set of values of variables (e.g. rate constants), we simulate 1000 individual model cells. From these simulations, we extract the mean and cell-to-cell variability in protein numbers in individual cells at steady state. We also estimate the mean activation time, defined as the time taken to reach half of the protein expression levels at steady state. Finally, as TFs and RNAP numbers only affect *τ*_prior_, we use *τ*_prior_/Δ*t* as a means to evaluate the effective influence of transcription initiation on the overall protein expression dynamics (as the dynamics of translation is identical in all models). We also explore how the features added by autoregulation, such as memory storage, bimodal activation and oscillations are affected by *τ*_prior_/Δ*t*, feedback strength and inducer concentration. We use this to determine the optimal parameter values for performing these tasks.

**Figure 2.**
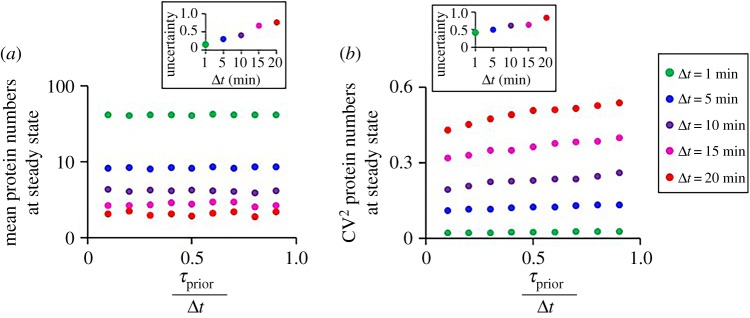
Dynamics of constitutive genes. (*a*) Mean protein numbers at steady state and (*b*) cell-to-cell variability in protein numbers at steady state for varying Δ*t* and *τ*_prior_/Δ*t*. The inset shows the uncertainty of the measurements in each condition.

We quantify noise in gene expression (variability in e.g. protein numbers over time) by the squared coefficient of variation (CV^2^) (squared mean over standard deviation). This quantity is shown to differ with *τ*_prior_/Δ*t* (e.g. figure 2*b*).

We further quantify the uncertainty (*U)* of estimation of a given quantity (*Q)* (e.g. CV^2^) from the simulations (due to this estimation being performed from a finite set of simulations). *U* is here quantified by the variance (equation (2.18)), and as expected is shown to differ with Δ*t* (i.e. usually, the higher is *Δ*t, the higher is *U*).

2.18 $$U=\frac{\mathrm{Standard}\;\mathrm{deviation}(Q)}{\mathrm{Mean}(Q)}.$$

To analyse oscillatory behaviours, we calculate the frequency (*F*) of the oscillations as follows, where *n* is the number of frequency bins in the spectrum, *F_i_* is the frequency of the spectrum at bin *i* of *n* and *Pxx_i_* = power spectral density (dB Hz^−1^) of spectrum at bin *i* of *n*:

2.19a $$F_{\mathrm{mean}}=\frac{\sum_{i=0}^{n}Pxx_{i}\cdot F_{i}}{\sum_{i=0}^{n}Pxx_{i}}\;.$$

We also calculate the spread of the amplitudes of each oscillation:

2.19b $$\mathrm{Spread}=\frac{\mathrm{Standard}\;\mathrm{deviation}(\mathrm{amplitude})}{\mathrm{Mean}(\mathrm{amplitude})}.$$

Finally, as noted, changing *k*_1_ in reaction 2.5 alters *τ*_prior_/Δ*t*. The resulting values of this parameter can be estimated from the rate constants of the models for constitutive, activated (auto- or externally) and repressed (auto- or externally) genes, as follows, respectively:

2.20 $$\frac{\tau_{\mathrm{prior}}}{\Delta t}=\frac{1}{1+\mathrm{RNAP}\cdot \;k_{1}/(k_{-1}+k_{2})},$$

2.21 $$\frac{\tau_{\mathrm{prior}}}{\Delta t}=\frac{1}{1+\;\mathrm{RNAP}\cdot k_{1}\cdot k_{B}/(\left(k_{B}+k_{U})\cdot (k_{-1}+k_{2}\right))}$$

2.22 $$\;\mathrm{and}\;\frac{\tau_{\mathrm{prior}}}{\Delta t}=\frac{1}{1+\;\mathrm{RNAP}\cdot k_{1}\cdot \;k_{U}/(\left(k_{B}+k_{U})\cdot (k_{-1}+k_{2}\right))}.$$

## 3. Results and conclusion

### 3.1. Transcription initiation kinetics affects the mean and noise in protein numbers at steady state, but not activation times of constitutive genes

Here, constitutive genes are used as a ‘null model’ to assess, by comparison, the effects of external and autoregulation by TFs. Thus, we first characterize the dynamics of this null model. We simulated the model in figure 1*a* for varying *τ*_prior_/Δ*t* (while keeping Δ*t* constant). Also, we changed Δ*t* for fixed *τ*_prior_/Δ*t* values. From the simulations, we extracted the mean and variability (as measured by CV^2^) of the protein numbers in individual cells at steady state, and the mean activation times. We also estimated the uncertainty in these variables (equation (2.18)). In these simulations, the model is initialized without proteins.

Results in figure 2 show that *τ*_prior_/Δ*t* does not affect the protein numbers at steady state (figure 2*a*), as expected, because Δ*t* was not altered. Only the cell-to-cell variability in protein numbers is affected, which is expected because higher *τ*_prior_/Δ*t* allows more frequent binding and unbinding of the RNAPs to the active promoters in between transcription events (figure 2*b*). As such, the uncertainty in these quantities is not affected (figure 2, insets). Meanwhile, changing Δ*t* while keeping *τ*_prior_/Δ*t* constant affects the mean and cell-to-cell variability of the protein numbers at steady state. Finally, the uncertainty in these quantities increases with Δ*t* (figure 2, insets), due to the decrease in mean RNA and protein numbers.

### 3.2. Rate-limiting steps in transcription initiation have different effects on autoregulated genes and externally regulated genes

**Figure 3.**
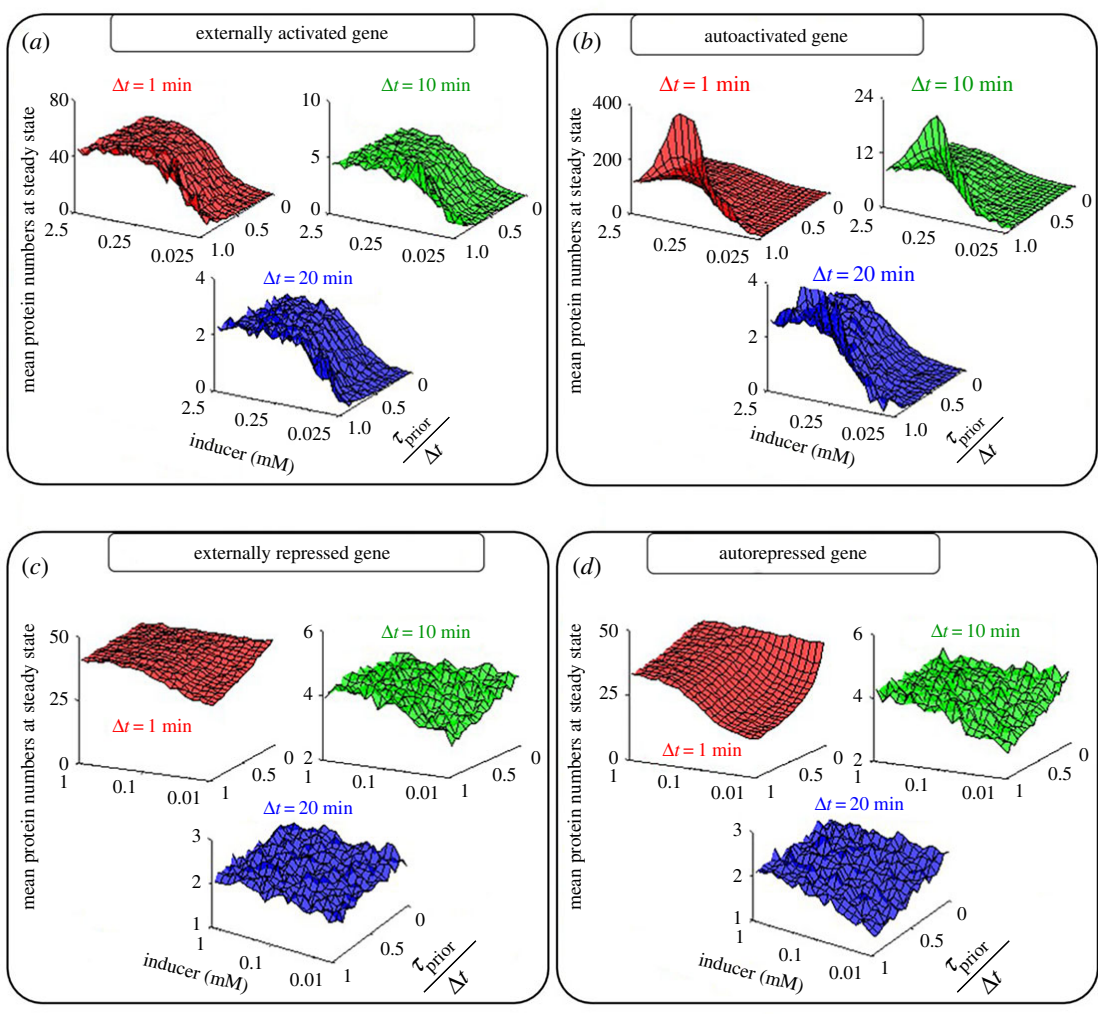
Mean protein numbers of externally regulated and autoregulated genes as a function of *τ*_prior_/*Δ*t and inducer concentration. (*a*) Externally activated, (*b*) autoactivated, (*c*) externally repressed and (*d*) autorepressed genes. Note the different scales in the axis showing the mean protein levels at steady state.

Previous studies showed that the sensitivity of a promoter's activity to TFs is affected by *τ*_prior_/Δ*t*, when TFs do not affect identically the kinetics of the rate-limiting steps in transcription initiation [18,23] (figure 1*f*). For example, consider two TFs with similar repressing capabilities, with one being able to double the mean duration of the first rate-limiting step, while the other can double the duration of the second rate-limiting step. In this scenario, if e.g. the first rate-limiting step is more longer-lasting than the second, the TF acting on the first step will have a stronger effect on the rate of RNA production. Thus, we hypothesized that the modes of regulation involving TFs (external and autoregulation) change in sensitivity with *τ*_prior_/Δ*t*. To test this, we changed inducer concentration and *τ*_prior_/Δ*t* and assessed the steady state expression levels in each model. Results are shown in figure 3.

Overall, in all cases, the quantitative behaviour of the circuits depends on all three variables (Δ*t*, *τ*_prior_/Δ*t* and inducer concentration). Meanwhile, the qualitative behaviour depends mostly on *τ*_prior_/*Δ*t and inducer concentration. Interestingly, the effects of each variable depend on the value of the other. E.g. in figure 3*b*,*d*, changing inducer concentration has stronger effects if *τ*_prior_/Δ*t* is large. Also, changing *τ*_prior_/Δ*t* has stronger effects for weak inducer levels (figure 3*d*).

In addition, comparing figure 3*a* and figure 3*b*, we find significant differences between external activation and autoactivation. Meanwhile, comparing figure 3*c* and figure 3*d*, we find little difference between external repression and autorepression. Further, comparing figure 3*a* and figure 3*c*, we see little difference between external activation and repression. Finally, comparing figure 3*b* and figure 3*d*, we see significant differences between autoactivation and autorepression.

Also from figure 3*a*,*b*, for weak inducer levels, decreasing *τ*_prior_/Δ*t* reduces protein numbers at steady state. This is due to the time window for the RNAP to bind to the unrepressed promoter being shorter. Externally activated genes change expression levels nearly monotonically with inducer concentration until reaching the plateau of full induction (figure 3*a,c*). Meanwhile, autoactivation causes this increase to be less monotonic (figure 3*b*). This would be relevant in the context of gene circuits, allowing sharper state shifting. Meanwhile, in figure 3*c*,*d*, for weak inducer levels, increasing *τ*_prior_/Δ*t* reduces protein numbers at steady state. I.e. it decreases leaky expression (i.e. protein production when in the presence of repressors).

### 3.3. Transcription initiation kinetics affects leaky expression in autorepressed genes

**Figure 4.**
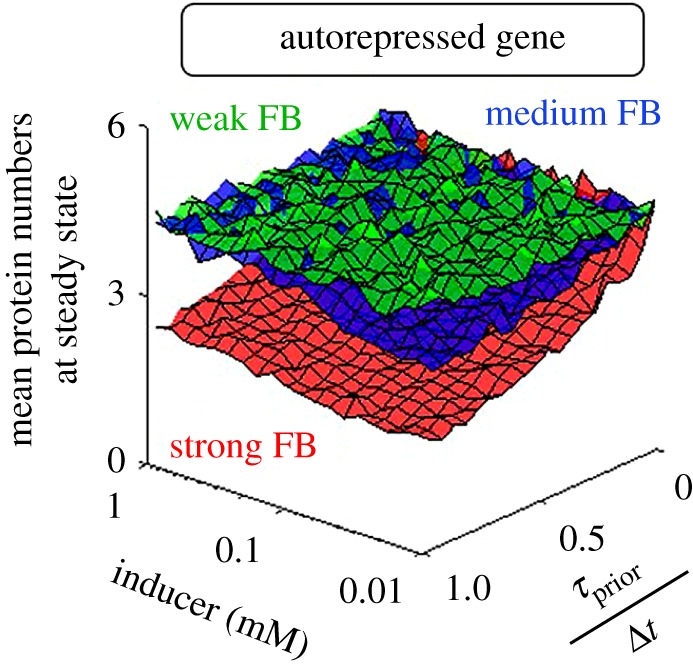
Steady state mean protein numbers of autorepressed genes for different autorepression strengths. Steady state protein numbers of autorepressed genes (Δ*t* = 10 min) as a function of inducer concentration and *τ*_prior_/Δ*t* for three levels of feedback strength. Weak, medium and strong FB (feedback strength) stand for *f* = 1/50, 1 and 50, respectively.

Repressed genes, especially autorepressed, exhibit leaky expression, which can be detrimental to cell growth rates [77–79] and facilitate fast state switching [80], etc. We investigate how leakiness can be tuned by *τ*_prior_/Δ*t* and autorepression strength. To change the latter, we alter the feedback strength, *f* (equation (2.3)). In figure 4, we show the steady state expression levels of autorepressed genes as a function of inducer concentration and *τ*_prior_/Δ*t* for three values of *f*.

From figure 4, weak feedback strength causes the circuit to be nearly impervious to changes in inducer concentration and *τ*_prior_/Δ*t*. Meanwhile, for strong feedback, protein numbers at steady state decrease with *τ*_prior_/Δ*t*. Owing to the negative autoregulation, induction levels have little to no effects. Overall, this suggests that increasing *τ*_prior_/Δ*t* along with strengthening the feedback strength is the best strategy for reducing leaky expression in autorepressed circuits.

### 3.4. Transcription initiation kinetics affects biphasic behaviour in autoactivation genes

**Figure 5.**
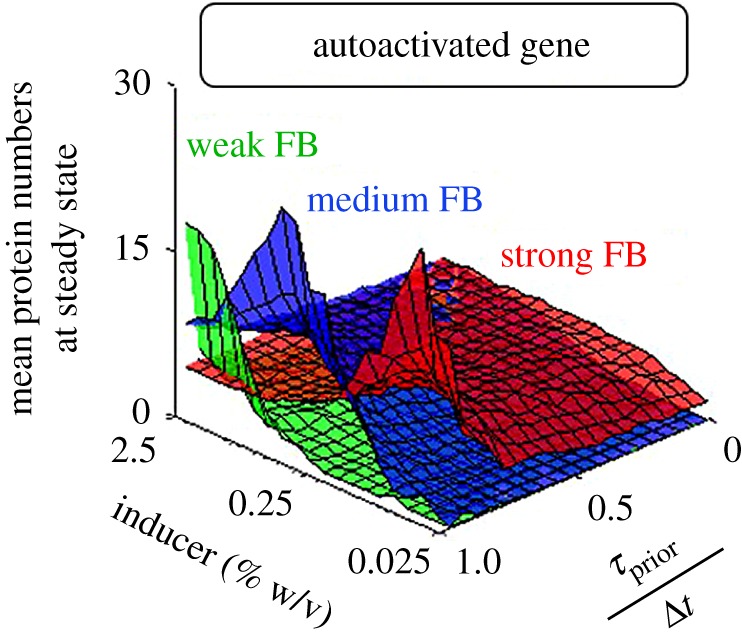
Steady state expression of mean protein numbers of autoactivated genes for different autoactivation strengths. The figure shows the steady state protein numbers of autoactivated genes (Δ*t* = 10 min) as a function of inducer concentration and *τ*_prior_/Δ*t* for three levels of feedback strength. Weak, medium and strong FB (feedback strength) stand for *f* = 1/50, 1 and 50, respectively.

From figure 3*b*, autoactivated genes exhibit biphasic behaviour for higher values of *τ*_prior_/Δ*t*. Next, we explore how to tune the threshold inducer concentration to reach biphasic behaviour as a function of *f* (equation (2.3)) and *τ*_prior_/Δ*t*. Results in figure 5 show the steady state expression levels of autoactivated genes as a function of these parameters.

**Figure 6.**
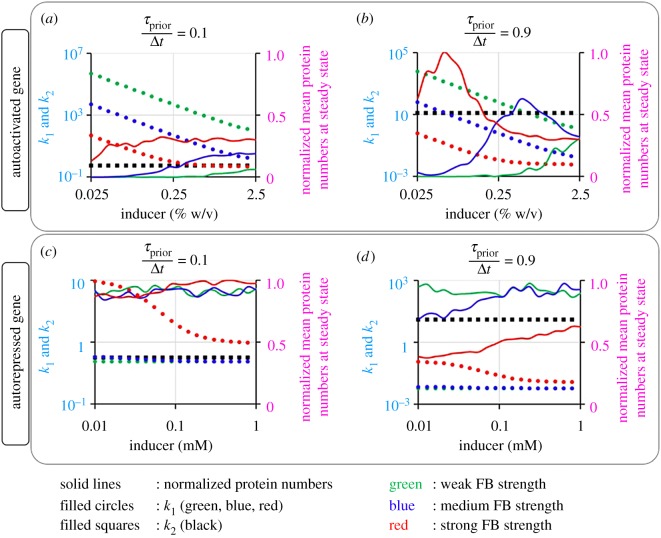
Kinetic parameters of autoregulated transcription allowing biphasic induction. The values of *k*_1_ and *k*_2_ of (*a*,*b*) autoactivated genes and (*c*,*d*) autorepressed genes were calculated for promoters with *τ*_prior_/Δ*t* = 0.1 and 0.9, under various induction levels and feedback strengths (low: *f* = 1/50, medium: *f* = 1 and high: *f* = 50). The values of *k*_2_ were represented by the black filled squares on the primary *y*-axis. The values of *k*_1_ were represented by filled circles (green, blue and red colours, indicating weak, medium and strong feedback strengths, respectively). Relative steady state expression levels were estimated from the simulations, normalized by the maximum expression level observed and are represented by curves on the secondary *y*-axis (green, blue and red colours corresponding to the weak, medium and strong feedback strengths, respectively).

From figure 5, stronger feedback allows the biphasic behaviour to occur at lower induction levels. Also, below a certain feedback strength, this phenomenon is no longer possible. The same occurs if *τ*_prior_/Δ*t* is too low. In this regard, figure 6*a*,*b* shows that having *k*_1_ > *k*_2_ allows higher expression levels as induction is increased (for high values of *τ*_prior_/Δ*t* alone), until a given threshold value, beyond which the opposite occurs, resulting in a biphasic behaviour. From figure 6*c*,*d*, this is not observed in autorepressed genes. Interestingly, the feedback strength determines the induction level at which the biphasic behaviour emerges. Overall, these results indicate that the kinetics of transcription initiation can play a key role in autoregulatory networks, even without affecting the mean transcription rate.

### 3.5. Transcription initiation kinetics affects cell-to-cell variability in protein numbers of autoregulated genes

**Figure 7.**
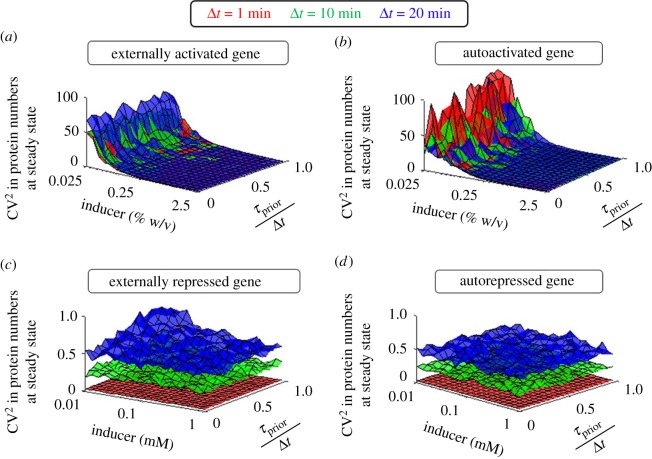
Cell-to-cell variability in the steady state protein numbers. (*a*) Externally activated, (*b*) autoactivated, (*c*) externally repressed and (*d*) autorepressed genes for three different values of Δ*t* and various inducer concentrations and values of *τ*_prior_/Δ*t*.

Next, we study how the kinetics of transcription initiation and the regulatory mode can be combined to regulate cell-to-cell variability in protein numbers in single-gene circuits with feedback. To quantify this variability, we use CV^2^ in protein numbers at steady state. Results are shown in figure 7.

From figure 7, in general, externally activated and autoactivated genes exhibit higher CV^2^ in protein numbers at steady state than externally repressed and autorepressed ones. Also, they are more sensitive to inducer concentrations. Externally activated and autoactivated genes differ in that the latter has more variability in behaviour. Meanwhile, in all cases (except in figure 7*c*, i.e. for externally repressed genes), *τ*_prior_/Δ*t* does not tune this variability significantly. Overall, we find the inducer concentration and, secondly, Δ*t* to be the main regulators of cell-to-cell variability in protein numbers at steady state, confirming again the hypothesis that the kinetics of transcription initiation can play a key role in autoregulatory networks, even without affecting the mean transcription rate.

### 3.6. Autoregulation allows transcription initiation kinetics to tune activation time

**Figure 8.**
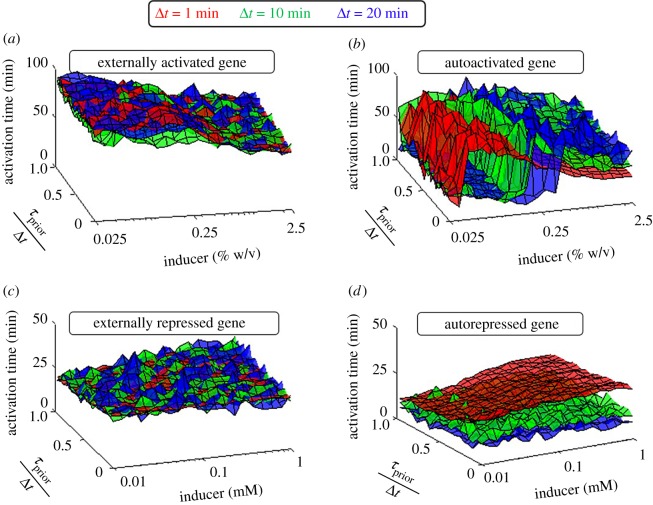
Activation time of externally regulated and autoregulated genes. The time to reach half the mean steady state expression levels of (*a*) activated, (*b*) autoactivated, (*c*) repressed and (*d*) autorepressed genes with different Δ*t* (represented in different colours) was estimated as a function of inducer concentration and *τ*_prior_/Δ*t*.

Precise timing of events is essential in complex cellular processes [81,82]. Typically, the expression levels of specific genes need to reach a threshold level to trigger subsequent events [81,83,84]. We studied how the inducer concentration and *τ*_prior_/Δ*t* affect activation times (here assumed to be the time to reach half the steady state level). Results are shown in figure 8.

In general, externally activated and autoactivated genes respond slower than externally repressed and autorepressed ones, in agreement with [85–87] (figure 8). Meanwhile, in all cases, activation times are nearly independent of *τ*_prior_/Δ*t*. Further, when and only when externally controlled, they are also nearly independent of Δ*t*. Finally, all circuits are affected by the inducer concentration.

### 3.7. Transcription initiation kinetics affects the memory storage capacity of autoactivated circuits

**Figure 9.**
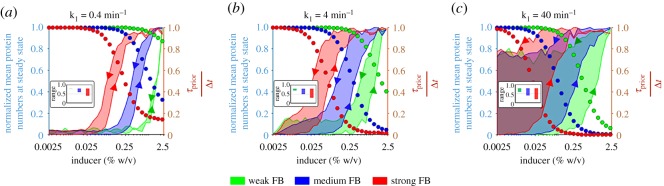
Memory storage capacity of autoactivated genes as a function of feedback strength and *τ*_prior_/Δ*t*. (*a*) *k*_1_ = 0.4 min^−1^, (*b*) *k*_1_ = 4 min^−1^ and (*c*) *k*_1_ = 40 min^−1^. In each, three circuits differing in feedback strength were simulated (green, blue and red colours represent weak, medium and strong feedback strength, respectively). The primary *y*-axis shows the normalized mean protein numbers at steady state and the secondary *y*-axis shows the estimated value of *τ*_prior_/Δ*t* (using equation (2.21)). Weak, medium and strong FB (feedback strength) stand for *f* = 1/50, 1 and 50, respectively.

We explore how the memory storage capacity of autoactivated circuits can be jointly tuned by the feedback strength (*f*) and the transcription initiation kinetics (*τ*_prior_/Δ*t* by varying *k*_1_, equation (2.21)). For this, for each case, cells were simulated under different induction levels until reaching their respective steady states (‘ON’ states). We studied the transition from the ON to the OFF state, by simulating cells whose initial condition corresponds to the steady state in protein numbers under full induction. For each model, two curves were generated, from OFF to ON, and from ON to OFF. Results in figure 9 show that, in all cases, the two curves do not overlap, demonstrating storage capacity for memory from past states.

From figure 9, increasing the feedback strength enhances the memory storage. Decreasing *k*_1_ reduces it. Namely, cells almost failed to store any memory when combining weak feedback with the smaller value of *k*_1_ tested (figure 9*a*). Surprisingly, the higher value of *k*_1_ caused the ON state to remain, even after removing the inducer (figure 9*c*). Finally, by changing *k*_1_ and the feedback strength, a wide range of values of *τ*_prior_/Δ*t* was covered (0.2 to 0.8). This range is reduced by weakening the feedback strength and/or *k*_1_ (figure 9, Insets).

### 3.8. Transcription initiation kinetics regulates the modality of cell populations with autoactivated genes

**Figure 10.**
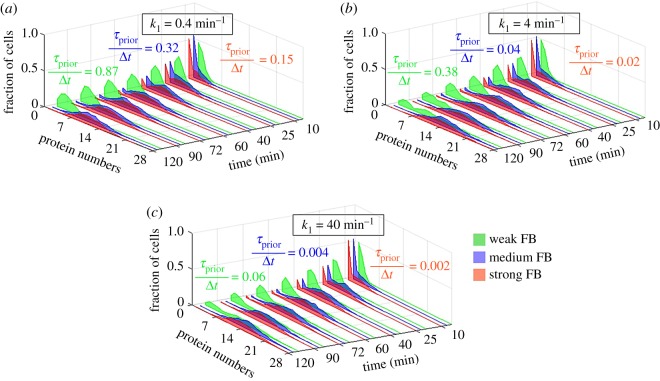
Bimodality in cell populations with autoactivated genes as a function of feedback strength and *τ*_prior_/Δ*t*. (*a*) *k*_1_ = 0.4 min^−1^, (*b*) *k*_1_ = 4 min^−1^ and (*c*) *k*_1_ = 40 min^−1^. Histograms of cells with various protein numbers at intermediate time points following the start of simulations. The values of *τ*_prior_/Δ*t* for each combination of feedback strength and *k*_1_ were estimated (using equation (2.21)). Weak, medium and strong FB (feedback strength) stand for *f* = 1/50, 1 and 50, respectively.

We study whether the behaviour of positively regulated genes can be jointly tuned by the transcription initiation kinetics (*k*_1_) and feedback strength (*f*). From the simulations, we obtained histograms of the fraction of cells with a given number of proteins at different time points (figure 10).

Figure 10 shows that the feedback strength can tune the probability of emergence of two ‘sub-populations’ from ‘one initial population’, as well as the fraction of cells in each sub-population. These are also affected by *τ*_prior_/Δ*t* (as regulated by *k*_1_). Meanwhile, the feedback strength affects the ranges of *τ*_prior_/Δ*t* that can be reached by tuning *k*_1_.

### 3.9. Transcription initiation kinetics affects oscillatory behaviour in autorepressed genes

**Figure 11.**
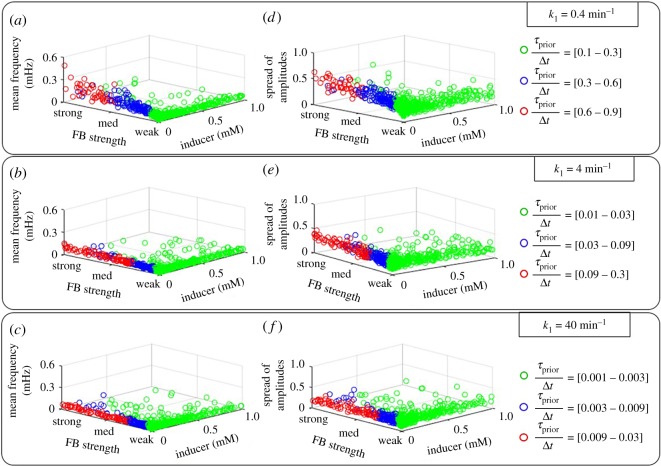
Oscillatory dynamics of autorepressed genes as a function of feedback strength and *τ*_prior_/Δ*t*. Autorepressed genes with different values of *k*_1_ (*k*_1_ = 0.4 min^−1^, *k*_1_ = 4 min^−1^ and *k*_1_ = 40 min^−1^) and feedback strengths were simulated, and the mean frequency of oscillations and the spread of amplitudes estimated from protein numbers over time. For each value of *k*_1_ and feedback strength, the values of *τ*_prior_/Δ*t* were calculated (using equation (2.22)). ‘FB’ stands for feedback strength, which is varied between *f* = 1/50 and 50.

Since *τ*_prior_/Δ*t* affects the time for transcript production to initiate, we hypothesize that it can be used to tune the dynamics of oscillations in the protein numbers resulting from autorepressed genes. We simulated these models for various values of *k*_1_ and feedback strength and extracted the mean frequency and spread (equation (2.19)) of the oscillations (figure 11). We also calculated the ranges of values of *τ*_prior_/Δ*t* (equation (2.22)) reached when changing the feedback strength and *k*_1_.

Results in figure 11 suggest an increase in both the mean frequency and spread of the oscillations for increasing feedback strength. This increase is sensitive to the value of *k*_1_. Overall, longer-lasting transcription initiation results in faster, more spread oscillations. Finally, in the regime of weak induction, *τ*_prior_/Δ*t* is mainly controlled by the feedback strength while, overall, the ranges of *τ*_prior_/Δ*t* decrease for increasing *k*_1_.

## 4. Discussion

The dissection of the dynamics of transcription initiation using *E. coli* as a model organism (first *in vitro* [14] and, more recently, *in vivo* [12,23]) has subsequently allowed showing that the kinetics of the rate-limiting steps in transcription initiation is sequence dependent, thus evolvable, and subject to external regulation, and thus adaptive. There is also much evidence that the kinetics of the two main rate-limiting steps can be tuned independently [11,18]. This has several consequences, e.g. two genes with similar rates of mRNA and protein production in one condition can differ widely in other conditions if the new conditions cause the relative durations of the rate-limiting steps of the two genes to differ (e.g. [13]). In recent works, it was also suggested that the effects of this phenomenon may have multi-scale effects, i.e. are tangible not only at the single-gene level but also at the level of small and large-scale genetic circuits [88–90].

Previous studies have thoroughly investigated how negative and positive regulation affects noise (e.g. [91]) and response times (e.g. [80]) in gene expression. Here, we focused on observing the state space of these complex small genetic circuits' models that combine autoregulatory mechanisms with rate-limiting steps in transcription. To assess the added value of autoregulation, the effects of a combined modification of the parameters of the multi-step transcription and the autoregulation on these circuits were compared to those in constitutive (used as null-models) and externally regulated genes.

Overall, we found that the efficiency with which the models exhibited complex dynamics regulation, such as minimization of leaky expression, biphasic behaviour, regulation of cell-to-cell variability in protein numbers, tuning of activation times, memory storage capacity and bimodal and oscillatory behaviour, was achieved by combining the tuning of the autoregulatory mechanism parameter values with the tuning of the rate-limiting steps in transcription.

This suggests that the strategy here used could be of assistance to improve the efficiency of presently existing synthetic genetic circuits. Relevantly, most predictions regarding the changes in kinetics obtained from the simulations could be tested by using such already engineered circuits (e.g. [46]), by changing their original promoters for others with different initiation kinetics (strength and, in particular, relative duration of the rate-limiting steps in transcription initiation [13,18,23]). Similar tests could be performed by changing the binding affinities of TFs to the promoters, whose original values can be found in [15,92–94], as these changes are expected to also allow changes in the transcription initiation kinetics of the genes composing the circuits.

While too extensive to introduce in the present work, in the future, it will be of interest to focus on specific models and further analyse how the various parameter values combine to generate the complex behaviours here reported, such as biphasic response and behavioural transitions.

Evidence suggests that prokaryotic cells evolved several autoregulated genes for time tracking, memory storage and decision making [29]. Given the results above, we hypothesize that this may have been made possible by co-evolving the transcription initiation kinetics of the component genes and the rate constants controlling the autoregulation.

In conclusion, our results may assist the engineering of single-gene synthetic circuits with predefined dynamics using the combined tuning of the feedback strength of the proteins and the kinetics of the rate-limiting steps in transcription initiation of the component promoter in order to maximize the circuit's efficiency. Such circuits, if efficient, may become of wide use due to their relative simplicity.

## Data accessibility

All relevant data, general methods and figures are within the main article. The Matlab codes are available on request from the authors.
